# Radioresistance in Hepatocellular Carcinoma: Biological Bases and Therapeutic Implications

**DOI:** 10.3390/ijms26051839

**Published:** 2025-02-21

**Authors:** Jianhui Wu, Xiaofang Zhao, Bowen Ren, Xuezhang Duan, Jing Sun

**Affiliations:** 1 Department of Radiation Oncology, The Fifth Medical Center of PLA General Hospital, Beijing 100039, China; docjianhuiwu@163.com (J.W.); hrhllj@163.com (X.Z.); 2 Medical School, Chinese PLA General Hospital, Beijing 100853, China; docrenbowen@163.com

**Keywords:** radiotherapy, radioresistance, hepatocellular carcinoma

## Abstract

Hepatocellular carcinoma (HCC) is a malignant tumor with high morbidity and mortality. Radiotherapy technology is a common treatment modality that can be used in all stages of HCC. However, in some cases, radiotherapy fails in clinical practice mainly because of the patient’s resistance to radiotherapy, creating a bottleneck for future breakthroughs. HCC radiosensitivity is primarily related to DNA double-strand break repair, cellular autophagy, cell cycle, cellular metabolism, and hypoxic environmental regulators. Therefore, a comprehensive understanding of its molecular mechanisms will be of immense importance in reversing HCC radioresistance. In this review, we provide a comprehensive overview of the mechanism of action of radiotherapy on HCC, the cellular and molecular basis of radiation resistance in HCC, related treatment modalities, and future prospects.

## 1. Background

Primary liver cancer is a common malignant tumor of the gastrointestinal system with a global mortality rate that ranks third among malignant tumors [1]. Radiotherapy is a common treatment method for hepatocellular carcinoma (HCC), a prevalent type of primary liver cancer [2]. This systemic, radical, or palliative treatment modality can be applied during all stages of HCC, regardless of the location, size, and stage of the lesion. The development of local precision radiotherapy technology has allowed the therapeutic dose of anti-cancer treatment to be concentrated in the target area of the tumor, achieving a high degree of conformal radiotherapy, which can protect healthy liver tissues and improve the efficacy of radiotherapy [3]. However, some patients are insensitive to radiotherapy, which manifests as in-field recurrence after treatment [3].

The “4R theory” of radiotherapy, suggested by Withers (1975), includes cell radiation damage repair, cell cycle redistribution, oxygen effect and hypoxic cell reoxygenation, and repopulation. This is the theoretical basis for prescribing radiation therapy doses and multiple radiation therapies. Later, Steel et al. added a fifth “R”, which is radiosensitivity, that is, radioresistance of tumor cells [4]. Radiotherapy resistance is the main reason for radiotherapy failure and is the starting point for exploring radiation resistance in HCC. Therefore, exploring the molecular mechanism underlying radiation resistance in HCC and finding potential therapeutic targets is essential for improving the efficacy of HCC radiotherapy and the prognosis of patients with HCC.

HCC is a hypermetabolic tumor fed mainly by the hepatic artery and requires more oxygen than surrounding normal hepatocytes, and uninhibited tumor cell proliferation often results in inadequate oxygen supply to surrounding tissues [5]. The rate of vascular proliferation in HCC is often mismatched with the rate of tumor volume increase and frequently combined with areas of intratumoral necrosis and abnormal vascular morphology, which lead to hypoxia within the tumor [5]. One study measured a median oxygen partial pressure of 30 mmHg in healthy livers, whereas in HCC, the median oxygen partial pressure was 6 mmHg [6]. The creation of hypoxic areas provides a microenvironment for HCC radioresistance [7]. In addition, multiple factors, including the DNA damage repair capacity of tumor cells, cell cycle alterations, autophagy and apoptosis, anti-oxidative stress system, and stroma and stem cells in the tumor microenvironment (TME), synergistically promote radioresistance development. Therefore, we reviewed the literature on the mechanism of action of radiotherapy on HCC, the cellular and molecular basis of radiation resistance in HCC, and related treatment modalities to recommend future possibilities.

This review systematically integrated key mechanisms—including DNA damage repair, autophagy, metabolic reprogramming, tumor hypoxia, and non-coding RNAs (ncRNAs)—to propose a comprehensive theoretical framework for understanding the molecular basis of radioresistance in HCC. Recent studies demonstrated that HCC radioresistance results from multifactorial interactions. Unlike previous research that primarily focused on the independent roles of single mechanisms, this review not only examined their individual contributions but also highlighted their complex synergistic effects. For instance, hypoxia-induced metabolic reprogramming enhances autophagy and DNA repair pathways, forming a positive feedback loop that significantly strengthens HCC radioresistance. Furthermore, ncRNAs regulate radioresistance through multiple pathways, such as modulating hypoxia-inducible factor-1α (HIF-1α) and its downstream signaling pathways as well as promoting autophagy to clear radiation-induced cellular damage. These processes maintain cellular homeostasis and further enhance resistance. These synergistic interactions reveal the intricate network underlying HCC radioresistance, providing novel insights into its molecular basis. By integrating these mechanisms, this study identified potential therapeutic targets for overcoming HCC radioresistance and proposes innovative treatment strategies. For example, combining radiotherapy with metabolic inhibitors (e.g., glycolysis-targeting agents), hypoxia-targeting drugs (e.g., HIF-1α inhibitors), and immunotherapy presents promising avenues for addressing HCC radioresistance. These strategies not only offer new directions for overcoming radioresistance but also establish a theoretical foundation for improving the clinical efficacy of radiotherapy in HCC.

## 2. Mechanisms of Radiotherapy

**Figure 1:**
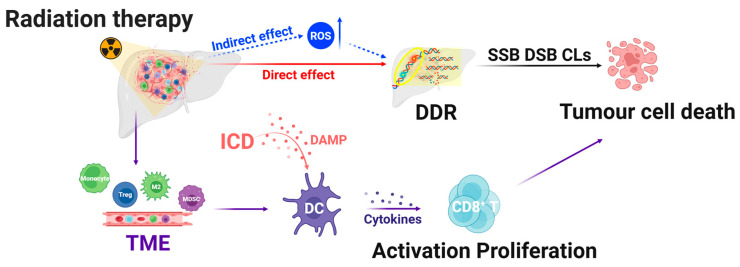
Mechanisms of radiotherapy. Radiotherapy induces tumor cell death through direct DNA damage, including SSBs, DSBs, and CLs, as well as indirect effects mediated by ROS, which activate the DDR. Radiotherapy triggers ICD, leading to the activation of DCs and CD8^+^ T cells. It also alters the TME by promoting hypoxia and recruiting immunosuppressive cells, such as Tregs, M2 macrophages, and MDSC, which may reduce therapeutic efficacy. ROS, reactive oxygen species; DDR, DNA damage response; SSBs, single-strand breaks; DSBs, double-strand breaks; CLs, clustered DNA lesions; DCs, dendritic cells; MDSC, myeloid-derived suppressor cells; TME, tumor microenvironment; ICD, immunogenic cell death; Tregs, regulatory T cells.

Radiotherapy is a main treatment modality for cancer that can destroy local cancer cells and tumor tissues via the direct and indirect action of high-energy photon radiation (including X-rays and γ-rays) while also modifying the vascular and immune systems in the area where the tumor is located (Figure 1) [8]. Radiation-induced endothelial cell dysfunction destroys blood vessels, aggravating hypoxia in the TME and inducing TME immunosuppression [9]. However, radiotherapy induces the immunogenic death of tumor cells and triggers anti-tumor immune responses [10].

### 2.1. Effects of Radiotherapy on Tumor Cells and Vascular System of HCC

Radiation-induced DNA damage response (DDR) is key for tumor elimination by radiotherapy, as it terminates tumor cell division and proliferation and induces necrosis and apoptosis mainly in the form of DNA double-strand and single-strand breaks. In addition to direct tumor elimination, radiation therapy can indirectly induce the production of reactive oxygen species (ROS), damage biomolecules, and alter cellular signaling pathways, such as inhibiting the PI3K/AKT/mTOR and PTEN signaling pathway or activating the P53 signaling pathway transduction, which induces cellular stress, ultimately resulting in cellular damage [11]. Furthermore, radiation disruption of angiogenesis increases the area of tumor hypoxia, reducing oxygen-dependent DNA damage and weakening the anti-tumor effect of radiation therapy while activating neointima formation, leading to post-irradiation cancer recurrence [12].

### 2.2. Effects of Radiotherapy on the Immune Microenvironment of HCC

Radiotherapy can modulate changes in the immune microenvironment of HCC. First, radiation upregulates the expression of major histocompatibility complex I molecules on the surface of tumor cells, promotes dendritic cell (DC) maturation and tumor-associated antigen presentation, enhances the secretion of cytokines required for T cell infiltration by DC and tumor cells, such as chemokine (C-X-C motifs) ligand 9 (CXCL9), CXCL10, and CXCL16, and expands CD8^+^ T cells to enhance their cytotoxic activity [13]. Second, radiation exposure upregulates FAS expression in tumor cells, enhances ligand binding to the CD8^+^ CTL surface, and induces immune-mediated cell death [14]. However, radiotherapy is a double-edged sword; it can activate immunosuppressive pathways and alter the HCC-TME into an immunogenic environment through the aforementioned mechanisms. For example, radiation damage to endothelial cells inhibits the infiltration of CD8^+^ T lymphocytes into the tumor and simultaneously induces the accumulation of immunosuppressive cells, including tumor-associated M2 macrophage, myeloid-derived suppressor cells, and regulatory T cells [15], which in turn produces resistance to radiotherapy and affects treatment efficacy.

### 2.3. Effects of Radiotherapy on Abscopal Responses of HCC

The abscopal effect refers to a phenomenon in which radiotherapy directly affects the local tumor site while simultaneously activating the immune system to inhibit or treat distant, non-irradiated tumor lesions [16]. This effect relies on radiotherapy-induced immunogenic cell death and associated immune activation mechanisms. In contrast, radiotherapy induces immunogenic cell death in tumor cells, during which a series of damage-associated molecular patterns, such as high-mobility group box 1 (HMGB1), calreticulin, and adenosine triphosphate, are released. These damage-associated molecular patterns act as potent immunostimulatory signals that activate DCs, enhance antigen presentation, and recruit and activate effector T cells to initiate innate and adaptive immune responses [17,18]. In contrast, high-dose radiotherapy can induce apoptosis or senescence of tumor cells through the p53 signaling pathway. These forms of cell death not only reduce tumor burden but also further release antigens and immunomodulatory factors, thereby enhancing the anti-tumor immune response [19,20]. In addition, exosomes serve as intercellular signaling tools and play a critical role in the abscopal effect [21]. Tumor cells treated with radiotherapy release exosomes carrying immune-activating molecules, such as inflammatory factors and tumor-associated antigens, which are transmitted to distant, non-irradiated tumor lesions through the circulatory system or the TME. These exosomes further amplify immune responses, significantly enhancing the systemic anti-tumor effects of radiotherapy [22].

## 3. Mechanisms of Resistance to Radiotherapy in HCC

Radiotherapy resistance is a multi-genetic and multi-mechanism process that includes anti-apoptosis through the activation of DNA damage repair for efficient DNA repair, promotion of cell survival through autophagy to provide energy and eliminate radiation-damaged proteins, modulation of cell cycle redistribution to reduce sensitivity to radiotherapy, increased glucose uptake by tumor cells, decreased mitochondrial oxidative phosphorylation, and a tumor hypoxic microenvironment induction of radiotherapy resistance.

### 3.1. DDR Pathway

**Figure 2:**
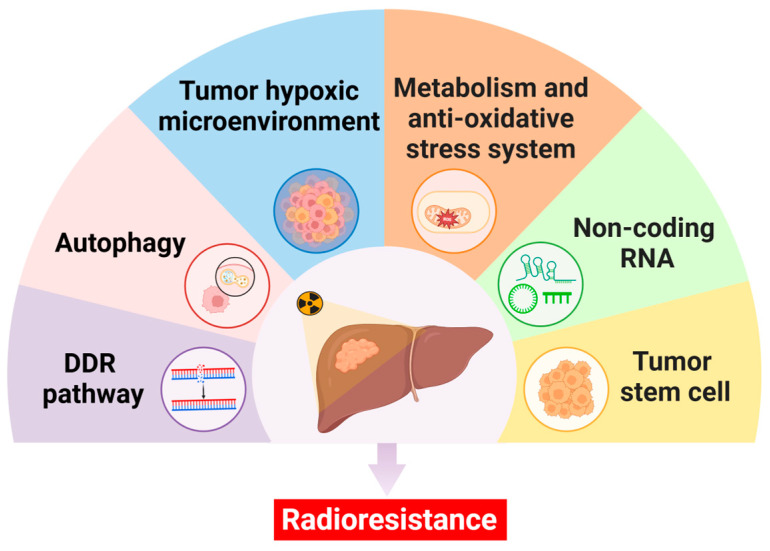
Molecular mechanisms of resistance to radiotherapy in hepatocellular carcinoma. Key mechanisms contributing to radioresistance include the DDR pathway, autophagy, the tumor hypoxic microenvironment, metabolism and anti-oxidative stress systems, non-coding RNAs, and tumor stem cells. These processes collectively enable tumor survival and limit radiotherapy efficacy. DDR: DNA damage response.

Radiotherapy damages the DNA of tumor cells directly or indirectly through ionizing radiation, causing cancer cell base destruction and DNA double-strand or single-strand breaks. The activation of DDR pathways after DNA damage, including DNA repair, cell cycle checkpoint activation, autophagy, and apoptosis, restores the DNA sequence structure and cellular homeostasis of tumor cells, leading to radiotherapy resistance (Figure 2) [23].

Methyltransferase 1 (METTL1) is a radiation resistance factor that protects HCC from ionizing radiation-induced DNA double-strand breaks and apoptosis. Researchers demonstrated this by utilizing in vitro models based on HCC cell lines (e.g., HepG2 and Huh7), xenograft models, and knockin and knockout mouse models, combined with advanced techniques such as m7G reduction and cleavage sequencing (TRAC-seq), polysome profiling, and polyribosome-associated mRNA sequencing. The findings indicated that METTL1-mediated m7G-tRNA modification can upregulate the translation of DNA-dependent protein kinase (DNA-PKcS) catalytic subunits or DNA ligase IV and promote double-strand break repair through the non-homologous end-joining pathway, leading to HCC radiotherapy resistance [24,25]. Another study found a high expression of proto-oncogene ubiquitin-binding enzyme E2T in HCC (UBE2T), which makes HCC less sensitive to radiotherapy. A study utilizing in vitro colony formation assays with HCC cell lines, xenograft tumor models, comet assays, cell cycle flow cytometry, γH2AX foci detection, chromatin fractionation, and immunofluorescence staining presented that UBE2T mediates H2AX/γH2AX mono-ubiquitination, maintains checkpoint kinase 1 (CHK1) activation, and provides sufficient time for radiation-induced DNA repair to confer radioresistance in HCC [26]. In addition, Jin et al. used small interfering RNA (siRNA) to inhibit the expression of survivin in HCC, which significantly increased the apoptosis rate of HCC cells after high-LET radiation, suggesting that survivin may be involved in radiotherapy resistance in HCC [27]. Radiotherapy can activate NF-κB, leading to the production of apoptosis-related and angiogenic factors that promote tumor cell survival and contribute to tumor growth [28]. Multiple studies revealed that inhibiting NF-κB activity can enhance the radiosensitivity of HCC cells. For example, lenvatinib suppresses Src/STAT3/NF-κB signaling, inhibits HCC cell proliferation, reduces invasion ability, and induces apoptosis, thereby enhancing radiosensitivity [29]. Furthermore, 18β-glycyrrhetinic acid improves the efficacy of radiotherapy in HCC by inhibiting EGFR/ERK/NF-κB signaling and promoting radiotherapy-induced apoptotic pathways [30]. The PI3K/AKT/mTOR signaling pathway is activated after DNA damage and closely relates to HCC radiotherapy resistance [31]. The application of PKI-587, a dual PI3K/mTOR inhibitor, was able to increase DNA damage, enhance G0/G1 cell cycle arrest, induce apoptosis, and increase the radiotherapy sensitivity of HCC [32]. HCC-derived RECQL4 reduces radiosensitivity by interfering with cGAS-STING pathway activation through DNA repair [33]. Preclinical studies by Bamodu et al. revealed that PDK1 drives radiotherapy resistance in HCC by activating the PI3K/AKT/mTOR signaling pathway, enhancing cancer stemness, and suppressing DNA damage, suggesting that targeting PDK1 could serve as a novel strategy to improve radiosensitivity [34].

### 3.2. Autophagy

In recent years, studies increasingly illustrated that autophagy plays a critical role in the radioresistance of HCC. GABARAP (γ-aminobutyric acid receptor-associated protein)-mediated autophagy and NEAT1v1-induced mitophagy are considered key mechanisms. By effectively clearing radiation-induced damaged components and restoring cellular homeostasis, these processes enhance the survival of HCC cells. GABARAP is involved in the formation and maturation of autophagosomes, playing a central role in the fusion of autophagosomes with lysosomes. NEAT1v1 promotes autophagy and radioresistance in HCC cells by upregulating GABARAP expression [35]. Specifically, knocking down NEAT1v1 using short hairpin RNA significantly downregulates GABARAP expression, inhibits autophagic activity, and increases the radiosensitivity of HCC cells. Conversely, overexpressing NEAT1v1 via plasmid DNA in a stable HCC cell model significantly upregulates GABARAP expression, enhances autophagic activity, and confers greater radioresistance to HCC cells. Knocking down GABARAP not only inhibits NEAT1v1-induced autophagy but also significantly enhances the radiosensitivity of HCC cells. This indicates that GABARAP-mediated autophagy protects tumor cells by clearing proteins and organelles damaged by radiation, thereby reducing structural and functional damage to cells [36].

In addition to participating in general autophagy through GABARAP, NEAT1v1 plays a critical role in radiation-associated mitophagy. Radiation induces oxidative stress and the accumulation of mitochondrial DNA damage, whereas NEAT1v1 enhances the resistance of HCC cells to radiation by regulating PINK1/parkin-mediated mitophagy [37]. The underlying mechanisms are as follows: X-ray radiation significantly increases oxidative stress and mitochondrial DNA content in HCC cells, but NEAT1v1 induces mitophagy to clear the damaged mitochondria, thereby suppressing oxidative stress and maintaining mitochondrial homeostasis. In HCC cells overexpressing NEAT1v1, the expression of parkin and superoxide dismutase 2 (SOD2) is significantly upregulated, and these molecules collectively reduce oxidative stress damage caused by radiation. Knocking down GABARAP or SOD2 impairs mitophagy, leading to the accumulation of damaged mitochondria and increasing the radiosensitivity of cells. NEAT1v1 promotes parkin expression by upregulating SOD2, whereas GABARAP accelerates parkin degradation through the mitophagy pathway. These findings highlight the unique role of NEAT1v1 in linking radioresistance to mitophagy. Moreover, molecular chaperone-mediated autophagy, a selective form of autophagy, reduces the levels of accumulated mutant p53 protein and contributes to tumor development. The radiation-induced activation of chaperone-mediated autophagy degrades HMGB1 protein and downregulates p53, thereby conferring radioresistance to HCC cells [38]. Autophagy is critical in protecting HCC cells from radiation-induced damage by effectively clearing proteins and mitochondria and maintaining cellular homeostasis. These findings underscore the importance of autophagy in the radioresistance of HCC and provide valuable insights into potential therapeutic targets for improving radiosensitivity.

### 3.3. Tumor Cell Metabolism and Anti-Oxidative Stress Systems

The development of radioresistance is associated with altered metabolism in tumor cells. Radiation-resistant tumor cells exhibit the Warburg effect, implying increased glucose uptake and decreased mitochondrial oxidative phosphorylation [39]. Inhibition of the Warburg effect may improve the sensitivity of tumors to radiation therapy (Figure 2).

Researchers screened radiation-resistant cell lines using a fractionated irradiation method with a linear accelerator. Specifically, MHCC97H cells were exposed to 2 Gy of irradiation daily for a total of 25 fractions, whereas MHCC97L cells received 8 Gy of irradiation every 2 days for a total of 5 fractions. After completing the irradiation regimen, the cells were allowed to recover for 4 weeks and subsequently subjected to a single dose of 10 Gy irradiation. They studied proteomics, metabolomics, and metabolic fluxes and observed that radiation-resistant HCC cells manifested an increased dependence on glucose. Increased glucose uptake promoted glucose synthesis into phospholipids, and the accumulation of phospholipids inhibited cytochrome c release and reduced radiation-induced apoptosis. Glucose addiction in HCC cells was dependent on HIF-1α and mTORC1-mediated radiation resistance in HCC by enhancing the translation of HIF-1α and SREBP1 [40]. The GSK-3β/AMPK/mTOR pathway is a signaling pathway that regulates glycolysis and is a target for radiosensitization. Based on in vitro models of HCC-LM3 and SK-Hep-1 hepatoma cells, along with assays for cell survival, migration, colony formation, DNA damage repair, and the expression of glycolysis-related proteins (e.g., GSK-3β, p-GSK-3β, AMPK, p-AMPK, mTOR, p-mTOR, GLUT-1/3, and PKM2), Huang et al. showed that the p-GSK-3β/GSK-3β ratio of HCC increased after radiotherapy. The traditional Chinese medicine extract osthole can inhibit the GSK-3β/AMPK/mTOR pathway and potentially weaken glycolysis, induce apoptosis, and increase the radiotherapy sensitivity of HCC [41]. Girdin, an actin-binding protein, is closely associated with tumor size and TNM stage in patients with HCC and is upregulated in HCC. Girdin may accelerate the proliferation, migration, and invasion of HCC cells. In addition, it was found that the radiation-induced apoptosis was significantly increased by silencing girdin in HCC cells. Further studies showed that girdin-short hairpin RNA increases radiotherapy sensitivity by inhibiting the PI3K-Akt pathway and glycolysis [42]. Hexokinase 2 (HK2) is highly expressed in radioresistant HCC cells and is associated with poor prognosis in patients. HK2 enhances radioresistance by forming a complex with the proapoptotic protein AIMP2 and promoting its autophagic lysosomal-dependent degradation, thereby attenuating radiation-induced apoptosis and promoting cell proliferation [43].

The KEAP1-NRF2-ARE signaling pathway is a key anti-oxidative stress system that plays a central role in HCC radiotherapy resistance. Under normal physiological conditions, NRF2 forms an inactive complex with its negative regulator, Keap1. Upon oxidative stress stimulation, NRF2 dissociates from Keap1, translocates into the nucleus, binds to the ARE, and upregulates downstream antioxidant gene expression. Sun et al. treated HepG2 (HCC cells) with the antioxidant isoliquiritigenin and inhibited NRF2 by enhancing keap1 expression in the antioxidant pathway, inducing apoptosis, and inhibiting tumor growth in HCC, thereby enhancing its sensitivity to radiotherapy [44]. In addition, γ-glutamylcysteine synthetase (γ-GCS) is a possible target for eliminating HCC radioresistance. Researchers, using a γ-GCSh-overexpressing cell line (GCS30 cells) model along with ROS detection, mitochondrial membrane potential assays, TUNEL assays, and HPLC techniques observed that γ-GCS is the rate-limiting enzyme that regulates glutathione biosynthesis and that γ-GCS can enhance the radiation resistance of HCC by catalyzing endogenous glutathione synthesis [45]. Nuclear protein 1 also plays a key role in regulating redox reactions in vivo. It is upregulated in HCC tissue and inhibits ROS production and oxidative stress through the AhR/CTP signaling axis, thereby enhancing cell viability during radiotherapy [46].

Ferroptosis is a form of cell death closely associated with tumor cell metabolism and anti-oxidative stress systems. RNA-seq and bioinformatics analyses have revealed that SOCS2 promotes the ubiquitination and degradation of SLC7A11 by recognizing its N-terminus through the SH2 domain, thereby inducing ferroptosis and enhancing radiosensitization. These findings suggest that targeting SOCS2 could improve the efficacy of radiotherapy in HCC and enhance patient prognosis [47]. Another study evaluated the expression and function of copper and copper metabolism MURR1 domain-containing protein 10 (COMMD10) using detection assays, immunostaining, radiation clonogenic assays, and 5-ethynyl-2′-deoxyuridine assays. The authors reported that COMMD10 enhanced ferroptosis and radiosensitivity in HCC by disrupting copper–iron homeostasis and inhibiting the HIF1α/CP loop [48]. Finally, the subcutaneous injection of the TLR3 agonist poly(I:C) during radiotherapy was found to not only activate tumor cell ferroptosis signaling but also enhance the abscopal effect, promote the DC-mediated presentation of tumor neoantigens, and increase the recruitment of activated CD8^+^ T cells to distant tumor tissues, thereby improving the radiosensitivity of HCC [49].

### 3.4. Tumor Hypoxic Microenvironment

HCC develops from hepatic cirrhosis caused by chronic liver injury, where persistent chronic inflammation leads to hepatic fibrosis and disruption of the normal hematological system. Insufficient hepatic blood circulation and high tumor proliferation ultimately lead to a local hypoxic microenvironment in HCC. Hepatic blood circulation can also participate in the regulation and stress response to the hypoxic microenvironment through the secretion of various factors and affect the radiotherapy sensitivity of HCC (Figure 2) [50].

HIF-1 is the major transcription factor specifically activated during hypoxia and comprises alpha (α) and beta (β) subunits in heterodimeric form, of which HIF-1α is the only oxygen-regulated subunit sensitive to oxygen partial pressure and determines HIF-1 activity. Under normoxia, von Hippel-Lindau ubiquitinates HIF-1α and causes its degradation. Under hypoxic conditions, the HIF-1α degradation pathway is blocked, stably expressed, and translocated to the nucleus, dimerizing with HIF-β to form the HIF-1 complex. Activated HIF-1 induces the expression of multiple genes whose products control angiogenesis, glucose metabolism, survival, and tumor spread [51]. HCC, one of the most hypoxic tumors, has high HIF-1 expression, and inhibition of HIF-1α expression reduces tumor cell proliferation, induces apoptosis, and enhances sensitivity to radiotherapy. Lai et al. illustrated that blocking the bFGF/PI3K/Akt signaling pathway (using pathway inhibitors LY294002 and naringenin) inhibited HIF-1α expression to reduce HCC radiotherapy resistance [52]. In addition, low concentrations of hydrogen sulfide may protect HCC cells from radiation damage. A study using sodium hydrogen sulfide and glibenclamide tablets (a selective adenosine triphosphate-sensitive potassium channel inhibitor) in concurrent treatment of HCC revealed that the radioprotective portion of hydrogen sulfide may be partially abrogated, demonstrating that hydrogen sulfide contributes to the increase in hypoxia-induced resistance to radiotherapy by opening adenosine triphosphate-sensitive potassium channels [53]. Shao et al. designed an innovative theranostic nanoplatform to reverse the hypoxic TME, promote a metabolic shift from glycolysis to oxidative phosphorylation, enhance sensitivity to cuproptosis, and significantly improve the radiosensitization of HCC [54].

The hypoxic microenvironment within HCC can lead to altered chromatin regulatory modifications in the associated tumor cells, affecting their radioresistance. A plant-derived polyphenol with anti-tumor properties reduced hypoxia-induced radioresistance in HCC by inhibiting JMJD2B [55]. Thus, the histone demethylase JMJD2B may be a druggable target for HCC radiosensitization. In a HepG2 cell model, SIRT1 induced c-Myc deacetylation under hypoxic conditions, reducing its accumulation and promoting HCC radioresistance. The use of nicotinamide, an inhibitor of SIRT1, enhances HCC radiosensitization [56].

### 3.5. Non-Coding RNA

ThEe ncRNAs are RNAs that do not code for proteins. The dysregulation of ncRNAs plays an important role in HCC development and regulates HCC radiosensitivity (Figure 2) [57]. Some ncRNAs positively regulate the radiosensitivity of HCC cells. The miR-146a-5p can inhibit proliferation and promote apoptosis by activating the DNA damage repair pathway and inhibiting the expression of replication protein A3 to increase radiosensitivity [58]. The miR-320b inhibits RAD21 expression by targeting RAD21 3′-UTR and increases γ-H2AX levels to improve the radiosensitivity of HCC [59]. The miR-621 is lowly expressed in HCC tissues. However, it targets the 3′UTR to inhibit SETDB1 expression, and the miR-621/SETDB1 signaling axis further activates the p53 signaling pathway and enhances radiosensitivity in HCC [60]. The miR-26b negatively regulates the expression of erythropoietin-producing human hepatocellular A2 protein and decreases the cell proliferation and migration rate of HCC after radiation, which acts as a sensitizer for radiation therapy [61]. The miR-203 enhances cell radiosensitivity in vitro and in vivo by targeting the B cell-specific Moloney leukemia virus insertion site 1 in HCC [62].

Researchers using miRNA database screening and RNA sequencing analyses revealed that miR-138-5p enhances the radiosensitivity of HCC by inhibiting EZH2 expression, downregulating H3K27me3 levels, and suppressing the HIF-1 signaling pathway, which collectively reduces cell migration, invasion, and epithelial–mesenchymal transition [63]. Similarly, miR-101-3p was found to enhance HCC radiosensitivity by directly targeting and suppressing WEE1 expression [64]. In another study, researchers transfected HCC cells (HepG2 and Huh7) with lentivirus carrying miR-122 and exhibited that miR-122 enhances radiosensitivity by inhibiting the mRNA and protein expression of Cyclin G1 [61]. Furthermore, through gene microarray screening of radiation sensitivity-related genes, combined with in vitro irradiation of HCC cells (HepG2 and MHCC97H), real-time PCR, and Western blot analyses, researchers identified that miR-22-5p promotes MIR22HG promoter histone acetylation by suppressing HDAC2 expression, which leads to the upregulation of MIR22HG expression and ultimately enhances HCC radiosensitivity [65]. The miR-621 enhances the radiosensitivity of HCC by directly suppressing SETDB1 expression and activating the p53 signaling pathway [60]. SLC16A1-AS1 promotes the radiosensitivity of HCC cells by targeting the miR-301b-3p/CHD5 axis [66]. Researchers using RNA immunoprecipitation, RNA pull-down assays, dual-luciferase reporter assays, and murine xenograft models exhibited that lncRNA GAS5 enhances the radiosensitivity of HCC by acting as a molecular sponge for miR-144-5p, thereby upregulating ATF2 expression [67]. Furthermore, the downregulation of lncRNA NEAT1_2 enhances the radiosensitivity of HCC cells by regulating the miR-101-3p/WEE1 axis [68]. In human HCC cells, knockdown of the hypoxia-inducible circular RNA ZNF292 increased sex-determining region Y-box 9 nuclear translocation, inhibited the Wnt/β-catenin pathway, and decreased hypoxic HCC cell proliferation and resistance to radiation therapy [69].

Many ncRNAs inhibit the efficacy of radiotherapy in HCC. One study reported that miR-193a-3p promotes DNA double-strand break repair to enhance HCC resistance to radiation [70]. In addition, miR-92b affects HCC’s response to radiotherapy. It is overexpressed in HCC tissues and cell lines and has been associated with poor patient prognosis. The overexpression of miR-92b promotes tumor cell proliferation, inhibits apoptosis, and ameliorates radiation-induced cell cycle arrest, which reduces the sensitivity of HCC cells to radiation [71]. In addition, in vivo and in vitro experiments manifested that the overexpression of miR-20a in HCC activated the PTEN/PI3K/Akt signaling pathway and induced radiation resistance in HCC cells [72]. Hypoxia induces miR-210 expression, which blocks tumor cells at the G0/G1 phase. The downregulation of miR-210 expression can effectively inhibit tumor cell survival, increase tumor cell apoptosis, and enhance radiotherapy sensitivity [73]. In addition, radiation treatment of SMMC7721 HCC cells under hypoxic conditions revealed the increased expression of lincRNA-p21. The knockdown of lincRNA-p21 resulted in G2/M phase blockage, increased apoptosis in HCC cells, and inhibited autophagy through the HIF-1/Akt/mTOR/p70S6K pathway, enhancing the sensitivity of HCC radiotherapy [74]. The lncRNA NEAT1 reduces the radiosensitivity of HCC through GABARAP-mediated autophagy regulation [35]. Similarly, the lncRNA LINC00473 decreases HCC radiosensitivity by regulating the miR-345-5p/FOXP1 axis [75]. Researchers, through the establishment of radioresistant cell lines, revealed that Linc-ROR acts as a competing endogenous RNA to regulate miR-145, thereby upregulating RAD18 expression, promoting DNA repair, and reducing HCC radiosensitivity [76]. Circular RNA is also involved in the mechanism of radiation resistance in HCC cells under hypoxic conditions. The circ-LARP1B was found to decrease HCC radiosensitivity by competitively binding to miR-578, which releases its repression on the target gene IGF1R [77].

### 3.6. Tumor Stem Cells Modulate HCC Radiation Resistance

Tumor stem cells comprise a small number of tumor cells with stem cell properties that reside in self-renewal, proliferation, and multidirectional differentiation potential and are relatively resistant to radiotherapy and chemotherapy, which are the root causes of tumor recurrence. The presence of tumor stem cells in primary HCC was confirmed with in-depth studies of the tumor stem cell doctrine, and CD133 is a tumor stem cell marker for radiotherapy and chemotherapy resistance. Piao et al. revealed that CD133-expressing HCC cells showed a higher activation of the MAPK/PI3K signaling pathway and lower ROS levels after radiation exposure, whereas results from a mouse transplantation tumor model showed that CD133-expressing HCC cells were resistant to apoptosis and radiation [78]. The 14-3-3ζ protein is widely involved in the regulation of cell cycle, signal transduction, and apoptosis. Lee et al. observed that the silencing of the 14-3-3ζ protein reduced the activity and number of hepatic tumor stem cells after γ-irradiation, induced apoptosis, and reduced the resistance of hepatic tumor stem cells to radiotherapy [79]. Zhu et al. designed an ROS-responsive liposome encapsulating the BMI1 inhibitor PTC-209 (LP(PTC-209)) and utilized ROS generated during radiotherapy as a stimulus to trigger its release. This release promoted the differentiation of cancer stem cells, restored the radiosensitivity of HCC, and successfully suppressed radioresistance [80].

## 4. Treatments

With a comprehensive understanding of radiation resistance mechanisms in HCC, new prospects for the radiosensitization treatment of HCC have emerged. Many mechanisms play a role in the response to ionizing radiation, and these include cell proliferation pathways, repair systems, apoptosis, and angiogenesis. Treatment based on these mechanisms can improve the radiotherapy sensitivity of HCC. In addition, improving the tumor’s hypoxic microenvironment by increasing the oxygen supply to the hypoxic areas of the tumor is an effective means of radiosensitization.

### 4.1. Targeting Relevant Molecules to Achieve Radiotherapy Hypersensitivity

Poly adenosine diphosphate-ribose polymerase 1 (PARP 1) is a DNA single-strand break repair enzyme that plays an important role in tumor cell death, differentiation, proliferation, as well as maintenance of genomic stability [23]. Many studies showed that PARP inhibitors (PARPi) enhance tumor cell sensitivity to radiation therapy in HCC models. Guillot et al. revealed that the use of Veliparib (PARPi) reduced HepG2 and PLC-PRF-5 cell survival and enhanced their radiosensitivity [81]. Subsequently, Gerossier et al. demonstrated that PARPi, in combination with radiation therapy, can enhance cytotoxicity, particularly in HBV-HCC [82]. The PARP inhibitor olaparib increases radiosensitivity and remodels the immune microenvironment by activating the cGAS-STING pathway, synergizing with radiotherapy to achieve local and distant tumor control [83]. Future clinical trials must evaluate the toxicity and potential benefits of radiotherapy in combination with PARP inhibition. Sood et al. reported a nanomaterial (alpha-ketoglutarate-decorated iron oxide-gold core-shell nanoparticles) that can increase the sensitivity of HCC to radiotherapy by increasing ROS generation and DNA fragmentation [84]. Zheng et al. reported that nanosilver and nanogold increase the level of DNA damage and apoptosis by upregulating Bax and caspase-3 expression and downregulating Bcl-2, catalase, superoxide dismutase, and total glutathione expression, thus enhancing the radiosensitivity of HCC [85].

Wnt/β-catenin and PI3K/AKT/mTOR signaling pathways play important roles in HCC radiotherapy resistance. Inhibiting survivin, the Wnt/β-catenin pathway target gene, with siRNA/cationic liposomes enhances the radiosensitivity of HCC [86]. Many studies revealed that inhibiting the PI3K/AKT/mTOR signaling pathway enhances the radiosensitivity of HCC [32,34,87]. MTOR proteins promote cell cycle G1/S progression and tumor proliferation as a primary function in the PI3K/AKT pathway and play an important role in HCC radiotherapy resistance. The mTOR inhibitors can act as novel radiosensitizers to improve the efficacy of HCC radiotherapy [88,89]. As mTOR blockers, combination radiotherapy with paromomycin or everolimus is a promising high-quality HCC treatment, and evidence-based medical studies are warranted in the future. Xie et al. found that nanoparticles containing lupeol can block DNA repair by inhibiting the activation of the PI3K/Akt pathway and MAPK phosphorylation in the Raf/MEK/ERK signal pathway, thus enhancing the radiotherapy sensitivity of HCC [87].

### 4.2. Improving the Hypoxic Microenvironment to Achieve Radiotherapy Hypersensitivity

HCC is a hypoxic solid tumor; thus, increasing the oxygen supply in the hypoxic region of the tumor is an effective means of sensitization by radiotherapy. In recent years, some scholars prepared nano-complexes to directly increase the oxygen supply to tumors, such as the hybrid semiconductor organosilicon-based nano-oxygen saver. The pHPFON-NO/O_2_ nano-complex interacts with the acidic TME to release NO to protect endogenous O_2_ while releasing O2 for exogenous O_2_ infusion [90]. Oxygen supply relies on hemoglobin in red blood cells. Therefore, increasing the oxygen transport capacity of the blood can improve the hypoxic microenvironment of tumors [91]. One study enhanced the radiosensitivity of hypoxic HCC by constructing self-assembled nanoparticles based on hemoglobin and curcumin by inhibiting cell proliferation, promoting apoptosis, and increasing DNA damage [92].

Targeting signaling and molecular expression within tumor cells to indirectly improve the hypoxic microenvironment of HCC is also a promising method for improving sensitization to radiotherapy. A research team introduced siRNA (or antisense oligonucleotide) to inhibit HIF-1α activity to weaken the invasion and metastasis of tumor cells and reverse the resistance of tumor cells to radiotherapy [93]. Epidermal growth factor receptor (EGFR) is highly expressed in most HCCs; thus, EGFR inhibitors can reduce VEGF expression and inhibit tumor angiogenesis by decreasing HIF-1α expression, thereby improving the hypoxic microenvironment and sensitivity to radiotherapy [94]. Multiple DNA damage signaling pathways in hypoxic tumor cells can cause radiation resistance, and the combined inhibition of cell cycle checkpoints and DNA repair targets is also helpful for improving radiotherapy resistance.

### 4.3. Harnessing the Abscopal Effect to Achieve Radiotherapy Hypersensitivity

After X-ray irradiation, tumor-associated antigen exposure in HCC cells increases, which simultaneously activates the body’s immune system [95]. Local radiotherapy not only directly kills tumor cells but also induces the abscopal effect, promoting systemic immune responses. Theoretically, this synergizes with immunotherapy to enhance the inhibition and elimination of tumor cells [96].

The combination of stereotactic body radiotherapy and immunotherapy can activate immune functions and induce the abscopal effect. In 1998, Ohba et al. reported a case of HCC with bone metastases. Following radiotherapy targeting a sternal metastatic lesion, not only did the local lesion shrink but the liver lesions also regressed spontaneously [97]. Additionally, increased serum TNF-α levels were observed after radiotherapy, suggesting that radiotherapy can activate the body’s anti-tumor immune response and induce the abscopal effect. Given its high-dose and highly effective biological dose characteristics, stereotactic body radiotherapy can cause rapid cancer cell death, releasing large amounts of tumor antigens, further stimulating the body’s anti-tumor immune response and enhancing the efficacy of immunotherapy. Nakabori et al. reported another case of advanced HCC with lung and adrenal metastases in which treatment with atezolizumab combined with bevacizumab was initially ineffective. However, after the addition of stereotactic body radiotherapy, followed by continued immunotherapy, the patient’s tumors significantly shrank, ultimately achieving complete remission [98].

Preclinical studies also support the potential of combining radiotherapy with immunotherapy to enhance anti-tumor responses. In multiple syngeneic mouse cancer models, low-dose fractionated radiotherapy was found to upregulate PD-L1 expression on tumor cells. Combining fractionated radiotherapy with PD-L1 inhibitors significantly improved local tumor control, prolonged survival, and provided protection against tumor re-invasion [99]. Radiation upregulates ALKBH5 in hepatic stellate cells, facilitating monocyte recruitment and M2 macrophage polarization. This forms a positive feedback loop that promotes radiation-induced liver fibrosis and reduces radiosensitivity in HCC. The dual roles of ALKBH5 as a microenvironmental regulator and a radiosensitization target offer new perspectives for preventing radiation-induced liver fibrosis and enhancing HCC radiosensitivity [100]. Maternal embryonic leucine zipper kinase (MELK) promotes HCC development, progression, and immune evasion by regulating the miR-505-3p/STAT3/CCL2 axis. Inhibiting MELK enhances radiotherapy-related immune effects, making it a potential target for molecular therapy and radiosensitization in HCC [101]. In addition, gut microbiome dysbiosis leads to primary resistance to radiotherapy in patients with HCC by suppressing anti-tumor immune responses mediated through the cGAS-STING-IFN-I pathway. This highlights the potential of gut microbiota modulation as a novel strategy for radiosensitization [102]. However, robust evidence from large-scale, randomized, controlled trials is lacking, and such trials are needed to confirm whether combining radiotherapy with immunotherapy can consistently improve clinical outcomes. Further studies are urgently needed to validate these synergistic effects and optimize specific treatment protocols.

## 5. Summary

HCC is a malignant tumor with the highest mortality rate of tumors globally, and advanced radiotherapy, such as stereotactic body radiation therapy, is a common treatment modality for suppressing localized HCC. However, improving sensitivity to radiotherapy is an urgent clinical challenge, and understanding the mechanism of radiation resistance occurrence in HCC is a prospective method for radiosensitization. Previous reviews on HCC radioresistance typically focused on individual mechanisms, such as DNA repair, cell cycle regulation, or the hypoxic microenvironment. In contrast, we adopted a more comprehensive approach in this review by integrating the interactions among multiple mechanisms, including DNA double-strand break repair, autophagy, metabolic regulation, the hypoxic microenvironment, and ncRNAs. Beyond merely discussing the roles of these individual mechanisms, this review highlights how their interplay collectively drives HCC radioresistance, providing a more holistic framework to guide future research in this field.

The PDK1/PI3K/AKT/mTOR, CAM/HMGB1/P53, Wnt/β-catenin, and Keap1/NRF2/ARE signaling pathways are closely related to HCC radiation resistance. Some studies confirmed that drugs targeting these pathways are helpful in increasing the radiotherapy sensitivity of other tumors. However, their sensitization to HCC radiotherapy has been inadequately studied, and future studies are required to fill this gap. In addition, inhibiting HCC metabolism is a promising therapeutic tool because HCC tumor cells exhibit the Warburg effect. Targeting key rate-limiting enzymes of tumor aerobic glycolysis is a key tool to improve the sensitivity of radiation therapy for laryngeal and non-small cell lung cancers, but this method needs to be fully investigated in HCC. Radiation therapy kills HCC tumor cells mainly through the “oxygen effect.” Therefore, the hypoxic microenvironment plays an indispensable role. Many studies reported that various novel biomolecular materials can improve the hypoxic microenvironment of tumors and enhance the killing of tumor cells. However, they were not studied extensively in HCC. HIF is a major regulator of the hypoxic microenvironment in HCC, and therapy that targets HIF is crucial for improving sensitization to HCC radiotherapy. However, this treatment has less preclinical data in HCC and requires further research. Recent studies predominantly focused on hypoxia-driven radioresistance and DNA repair as separate mechanisms. However, this review emphasizes the interplay between the two, illustrating how the hypoxic microenvironment not only reprograms cellular metabolic states but also upregulates DNA damage repair pathways, thereby intensifying radioresistance. By integrating these associations, this review offers novel perspectives for the development of combined therapeutic strategies in the future.

The regulatory roles of ncRNAs have been increasingly reported on; however, previous studies were predominantly experimental and lacked systematic summarization. This review, for the first time, consolidates advancements in ncRNA research and proposes how they play a central role in HCC radioresistance by regulating multiple signaling pathways, such as DNA repair and cellular metabolism. Furthermore, it explores the potential of ncRNAs as therapeutic targets and recommends utilizing multi-omics approaches, such as single-cell sequencing and metabolomics, to further investigate the mechanisms of radioresistance. In particular, integrating ncRNA regulatory networks could help identify more effective intervention targets.

This review provides a comprehensive overview of the key studies on HCC radioresistance published in the past 5 years and highlights unresolved challenges in the field. Current research on HCC radioresistance primarily relies on in vitro or animal models, with several mechanisms yet to be validated in clinical trials. Future research should focus on strengthening in vivo experiments to further validate the mechanisms of radioresistance and related intervention strategies. Additionally, findings should be confirmed in larger, multi-center cohorts to enhance the reliability and clinical applicability of the results. Concurrently, future studies should investigate other molecular pathways potentially associated with radioresistance, such as immune evasion, tumor metabolic reprogramming, ncRNA regulation, and the role of the TME. These investigations will provide additional potential targets for developing novel therapeutic strategies. Patients with HCC often present with significant heterogeneity, including differences in pathological types, liver function status, and underlying conditions such as viral hepatitis and liver cirrhosis. In particular, patients with decompensated cirrhosis have poor liver function, which can greatly impact radiotherapy tolerance and efficacy. This heterogeneity may limit the generalizability of current findings. Future studies should stratify patient populations based on pathological type, liver function, and viral hepatitis background and validate findings in larger, multi-center cohorts to improve their robustness and applicability. In addition, combined treatment strategies, such as radiotherapy combined with interventional therapies, targeted therapies, or immunotherapies, require further optimization. These strategies must account for liver function and tolerance to ensure safety and efficacy. Multidisciplinary collaboration involving experts in oncology, radiotherapy, and hepatology is critical for developing individualized treatment plans. Furthermore, novel radiosensitizers should be developed and evaluated, particularly for targeting molecular pathways associated with radioresistance. Fundamental and translational research plays a critical role in bridging the gap between experimental discoveries and clinical applications, ensuring that these novel therapies effectively enhance the efficacy of radiotherapy for HCC. However, despite promising findings from basic research, the clinical application of radiosensitizing drugs remains a lengthy and complex translational process. Specifically, radiosensitization strategies targeting particular mechanisms—such as hypoxia or metabolic reprogramming—must undergo rigorous preclinical validation and be further assessed through clinical trials to evaluate their safety, efficacy, and cost effectiveness. Moreover, several obstacles must be addressed for practical implementation, particularly in resource-limited settings, including the need for specialized training, inadequate infrastructure, and financial constraints. Therefore, future research should prioritize overcoming these challenges and seek solutions through multidisciplinary collaboration to ensure that innovative therapeutic strategies can be seamlessly integrated into real-world healthcare systems, ultimately benefiting a broader patient population.

In conclusion, the resistance mechanism to radiotherapy in primary HCC is highly complex, with multiple regulatory factors involved and various factors interacting. The mechanism of radiotherapy resistance in primary HCC requires more in-depth study and exploration and will be of great significance for the selection of patients viable for radiotherapy, finding individualized radiotherapy doses and target areas, and providing guidance for developing novel radiotherapy sensitizing drugs.

## Abbreviations

AMPK, AMP-activated protein kinase; ARE, antioxidant response elements; CLs, clustered DNA lesions; CXCL10, C-X-C motif chemokine ligand 10; CXCL16, C-X-C motif chemokine ligand 16; CXCL9, C-X-C motif chemokine ligand 9; DC, dendritic cell; DDR, DNA damage response; DSB, double-strand break; EGFR, epidermal growth factor receptor; ERK, extracellular signal-regulated kinase; GSK-3β, glycogen synthase kinase-3β; HCC, hepatocellular carcinoma; HK2, Hexokinase 2; HIF-1, hypoxia-inducible factor-1; HMGB1, high-mobility group box 1; KEAP1, Kelch-like ECH-associated protein 1; MDSC, myeloid-derived suppressor cells; MELK, maternal embryonic leucine zipper kinase; METTL1, methyltransferase 1; mTOR, mammalian target of rapamycin; ncRNA, non-coding RNA; NF-κB, nuclear factor kappa B; NRF2, nuclear factor erythroid 2-related factor 2; PARPi, poly adenosine diphosphate-ribose polymerase inhibitor; PDK1, pyruvate dehydrogenase kinase 1; ROS, reactive oxygen species; siRNA, small interfering RNA; SSB, single-strand break; TLR3, Toll-like receptor 3; TME, tumor microenvironment; UBE2T, ubiquitin-binding enzyme E2T; γ-GCS, γ-glutamylcysteine synthetase.
